# Blockade of endothelin-1 receptor B regulates molecules of the major histocompatibility complex in sickle cell disease

**DOI:** 10.3389/fimmu.2023.1124269

**Published:** 2023-02-28

**Authors:** Yaritza Inostroza-Nieves, Alicia Rivera, José R. Romero

**Affiliations:** ^1^ Division of Endocrinology, Diabetes and Hypertension, Department of Medicine, Brigham and Women’s Hospital, and Harvard Medical School, Boston, MA, United States; ^2^ Department of Laboratory Medicine, Boston Children's Hospital, Boston, MA, United States; ^3^ Department of Biochemistry and Pharmacology, San Juan Bautista School of Medicine, Caguas, Puerto Rico; ^4^ Department of Pathology, Harvard Medical School, Boston, MA, United States; ^5^ Division of Nephrology and Vascular Biology Research Center, Beth Israel Deaconess Medical Center, Boston, MA, United States

**Keywords:** major histocompatibility complex, endothelin-1, sickle cell disease, endothelin-1 receptor B, endothelin-1 receptor antagonist BQ788

## Abstract

Major Histocompatibility Complex (MHC) molecules have been proposed to play a role in Sickle Cell Disease (SCD) pathophysiology. Endothelial cells express MHC molecules following exposure to cytokines. SCD is characterized, in part, by vascular endothelial cell activation, increased oxidative stress, sickle cell adhesion, and excess levels of endothelin-1 (ET-1) contributing to vaso-occlusive crises. ET-1 activates endothelial cells, induces oxidative stress and inflammation, and alters erythrocyte volume homeostasis. However, the role of ET-1 on MHC regulation in SCD is unclear. We first studied two sickle transgenic knockout mouse models of moderate to severe disease phenotype, βS-Antilles and Berkeley (BERK) mice. We observed significant increases in H2-Aa mRNA levels in spleens, lungs, and kidneys from transgenic sickle mice when compared to transgenic knockout mice expressing human hemoglobin A (HbA). Mice treated for 14 days with ET-1 receptor antagonists significantly reduced H2-Aa mRNA levels. We characterized the effect of ET-1 on MHC class II expression in the human endothelial cell line EA.hy926. We observed dose-dependent increases in the expression of MHC class II (HLA-DRA) and MHC transcription factor (CIITA) that were significantly blocked by treatment with BQ788, a selective blocker of ET-1 type B receptors. Chromatin immunoprecipitation studies in EA.hy926 cells showed that ET-1 increased Histone H3 acetylation of the HLA-DRA promoter, an event blocked by BQ788 treatment. These results implicate ET-1 as a novel regulator of MHC class II molecules and suggest that ET-1 receptor blockade represents a promising therapeutic approach to regulate both immune and vascular responses in SCD.

## 1 Introduction

Intravascular erythrocyte sickling leads to vaso-occlusion and ischemic injury and drives many adverse clinical manifestations of Sickle Cell Disease (SCD) (1). However, there is growing concern that patients with SCD show evidence of immune dysfunction and increased susceptibility to invasive bacterial infections (2–4). In children and adults with SCD, viral infections such as influenza and H1N1 have been reported to be associated with increased pulmonary complications, including acute chest syndrome (ACS) and pain requiring intensive care (5, 6). As such, it has recently been proposed that patients with SCD are at increased risk for developing ACS and respiratory complications following severe acute respiratory syndrome coronavirus-2 (SARS-CoV-2) infection (7).

Immune cells, such as monocytes, neutrophils, and platelets, play an essential role in the pathophysiology of SCD (8, 9). Several reports have revealed an association between white blood cell (WBC) counts and the severity of complications in SCD (10–13). Yousif reported that elevated leukocyte count was responsible for worse clinical manifestations in SCD (14). These patients showed increased levels of circulating C-reactive protein, inflammatory cytokines, activated neutrophils, and von Willebrand factor antigen, which suggest chronic endothelial cell activation (15). Activated endothelial cells express increased levels of major histocompatibility complex (MHC) class II molecules, adhesion molecules, and chemokines, further contributing to inflammatory responses (16).

In SCD, activated endothelial cells release the proinflammatory and vasoactive peptide endothelin-1 (ET-1) (17). ET-1 is a potent vasoconstrictor that we and others have shown is an important contributor to the hematological, vascular, and renal complications of SCD (18–20). ET-1 stimulates the release of proinflammatory cytokines and molecules, including reactive oxygen species and extracellular protein disulfide isomerase, stimulating erythrocyte KCNN4 activity, leading to modifications of the vascular wall (21) and erythrocyte volume (20, 22). We documented that *in vivo* treatment of SCD mouse models with ET-1 receptor antagonists (ETRA) led to significant improvements in hematological parameters and red cell hydration status (20, 23, 24). Also, ET-1 increases epithelial permeability allowing antigen translocation (25), and ETRAs reduce the levels of MHC class II molecules in a rodent model of renal allograft dysfunction (26). In addition, increased ET-1 has been proposed to play a role in organ transplant rejection. As such, ETRAs have been suggested as novel therapeutic approaches to reduce transplant failure rates (27).

MHC molecules play a vital role in immune responses, organ transplants, and the progression of vascular inflammation (28–30). MHC class II molecules are cell-surface glycoproteins in antigen-presenting cells, B cells, and endothelial cells, among others, that bind and present extracellular antigenic peptides. Six main MHC class II genes that are part of the human leukocyte antigen (HLA) have been described in humans. Of these MHC class II genes, there is evidence that genetic variants of HLA-DPB1 are associated with an increased risk of stroke (31, 32) and HLA-DRB1*100101 is associated with vaso-occlusion (33) in patients with SCD. In addition, polymorphic gene variants in HLA-DPB1 and HLA-DQB1.h are associated with an increased risk for severe infections in patients with SCD (34). However, the relationship between ET-1 and MHC class II molecules in SCD remains unclear. We hypothesized that ET-1 would increase MHC expression through the activation of ET-1 receptors and alter MHC responses in SCD. In this study, we investigated the role of ET-1 on MHC class II molecules in transgenic SCD mice and endothelial cells.

## 2 Methods

### 2.1 Animals

We used the Berkeley (BERK) sickle cell transgenic mouse model that was developed on a C57BL/6J background (The Jackson Laboratories, Bar Harbor, ME, USA) as described by us and others (20, 35). BERK mice contain normal human α-, γ-, and δ-globins and sickle β-globin as well as targeted deletions of murine α- and β-globins. BERK mice have severe disease that simulates human sickle cell anemia (hemolysis, reticulocytosis, anemia, extensive organ damage, and shortened life span) and has high levels of oxidative stress (35). As controls for our mouse studies, we used transgenic mice expressing exclusively normal human hemoglobin A and knockout mouse globins (HbA) (36). The βS-Antilles transgenic mice were kindly provided by Dr. Mary Fabry (Albert Einstein College of Medicine, Bronx, NY, USA). All procedures for study, animal care, and euthanasia followed approval by the Boston Children’s Hospital Animal Care and Use Committees.

### 2.2 *In vivo* studies

We studied HbA, BERK, and βS-Antilles male mice between 8-12 weeks of age placed on an endothelin-1 receptor antagonist mix (ETRA) regime for 14 d, as previously reported by us in SAD mice (20, 23, 24). Briefly, mice were intraperitoneally injected for 14 consecutive days and received either sterile mouse saline (0.1 ml) or 0.1 ml of an ETRA mixture that consisted of selective ET-1 antagonist subtype A (BQ123; 0.2 mg/ml) and selective ET-1 antagonist subtype B (BQ788; 0.2 mg/ml) dissolved into 1 ml mouse saline. Animals were fed standard mouse chow and given water ad libitum during treatment. Mice were then euthanized, and whole blood was immediately collected into heparinized or EDTA tubes for ET-1 plasma level measurement. Whole blood cell counts were performed in EDTA-collecting tubes and analyzed by ADVIA 120TM hemoanalyzer. Spleen, lung, and kidneys were carefully excised, gently blotted, weighed, and stored at -80°C in RNAlater™ Stabilization Solution (Thermo Fisher Scientific, Waltham, MA USA).

### 2.3 Laboratory methods

#### 2.3.1 HPLC measurement of ET-1 plasma levels

ET-1 plasma levels from HbA, BERK, and βS-Antilles were determined by HPLC as described previously (23). Briefly, blood samples were collected in Eppendorf tubes containing 10mg/mL EDTA, 2 mM PMSF, and 500 µg/mL aprotinin. Blood was centrifuged at 2500rpm for 10 min. Plasma was isolated and added to 600 µl of acetone mixture (40 acetone:1 (1N) HCl:5 water). The sample was dried under nitrogen and reconstituted with 75 µL of Mixture A (30% acetonitrile in 0.1% Trifluoroacetic acid TFA). The extracted samples were transferred to HPLC vials for analysis.

HPLC unit: Supelcoil LC-318 reverse–phase column (25cm length, 4.6 mm id, 5 µm particle size, 300Å, Supelco, Oakville, ON), fluorescence detector RF551 Shimadzu and a temperature control chamber. ET-1 was resolved using gradient elution with two solvents, Mixture A and Mixture B (90% acetonitrile in 0.1% TFA), with a 20 µl injection volume and the mobile phase set at 1ml/min.

#### 2.3.2 Cell culture

Human endothelial cells, EA.hy926, were maintained in completed Dulbecco’s modified Eagle’s medium (DMEM) with 10% fetal bovine serum (FBS) and 1% Penicillin/Streptomycin as previously described by us (36, 37). These cells showed positive staining for endothelial cell markers as they were positive for VWF and CD31 using immunofluorescence techniques and expressed caveolae as previously described (37).

#### 2.3.3 Gene expression - quantitative RT-PCR

To measure the relative MHC class II gene expression, RNA from mouse spleen, lung, and kidney or EA.hy926 cells were prepared using Trizol and reversed transcribed using the Superscript II kit (Invitrogen, Carlsbad, CA, USA), as described by us (20, 37, 38). Briefly, TaqMan Universal PCR master mix and TaqMan primers for H2-Aa, HLA-DRA, CIITA, β-2-microglobulin and GAPDH (Applied Biosystems, Foster City, CA, USA) were used for the real-time PCR. The cycle threshold (Ct) values for H2-Aa, HLA-DRA, and CIITA expression were normalized against two endogenous controls, β-2-microglobulin and GAPDH. The 2−ΔΔCt method was used to analyze the difference in mRNA expression between ETRA-treated and vehicle-treated sickle mice and vehicle and ET-1-treated cells (39).

#### 2.3.4 Chromatin immunoprecipitation (ChIP)

EA.hy926 cells were plated at a density of 1x106 cells/mL and were treated with or without 100 nM ET-1 for 24h to study the association of MHC class II transcription factors with MHC class II promoters. After treatment, cells were crosslinked with 1% formaldehyde for 10 min at room temperature. Glycine was used to stop the crosslinking reaction. We performed the assay according to the manufacturer’s instructions (EZ-Magna ChIP HiSens Kit from Millipore). Briefly, nuclei were isolated and lysed using the single buffer system (SCW) buffer. Chromatin was sheared using a Misonix 3000 with 12 pulses at 65% power to generate an average of 100 – 500 base pairs of sheared DNA. Protein A/G beads were used to pre-cleared the sonicated lysates and then immunoprecipitated with antibodies against Histone 3 (H3), acetyl-H3 (Lys 9), CREB (from Millipore), CIITA (Santa Cruz Biotechnology) or Rabbit IgG overnight at 4°C. Samples were washed for 5 min at 4°C with the SCW buffer and Low Stringency Buffer. Samples were eluted with ChIP Elution Buffer. Finally, the crosslink was reversed with 5 M NaCl at 65°C for 2 h, and immunoprecipitated DNA was analyzed by real-time PCR (StepOne Plus, Applied Biosystems).

#### 2.3.5 Measurement of total and phosphorylated levels of CREB

We measured the total and phosphorylated levels of Cyclic AMP-responsive element-binding protein (CREB) to analyze the effect of ET-1 treatment on phosphorylation of CREB. EA.hy926 cells were collected and homogenized in lysis buffer, and protein concentration was determined using the DC Protein Assay from Bio-Rad. Analyses were performed using monoclonal antibodies against CREB and phosphorylated CREB protein using the InstantOne CREB (Total/Phospho) ELISA Kit (purchased from Thermo Fisher Scientific) according to the manufacturer’s protocols. The results are expressed as fold changes relative to the vehicle, representing the total baseline and phosphorylated CREB levels.

### 2.4 Statistical analysis

Results are expressed as means ± SD unless otherwise stated. GraphPad software (Prism 9 version) was used to evaluate statistical significance among animal groups. Analysis was done using paired (Wilcoxon) or unpaired (Mann-Whitney) nonparametric tests. Significance was set at P < 0.05.

## 3 Results

### 3.1 Treatment of SCD mice with endothelin-1 receptor antagonists leads to reduced expression of MHC class II molecules in the spleen, lungs, and kidneys

Increased ET-1 levels and erythrocyte hydration status play essential roles in SCD through unresolved mechanisms. As previously reported in SCD mice, plasma ET-1 levels were significantly elevated in BERK (325 ± 31 pmol/mL, n=3, p<0.001) and βS-Antilles (211.9 ± 128 pmol/mL, n=3, p<0.01), relative to HbA mice (123 ± 25 pmol/mL, n=3) (23). These results are consistent with previously reported findings in human plasma from sickle cell patients (40).

**Figure 1:**
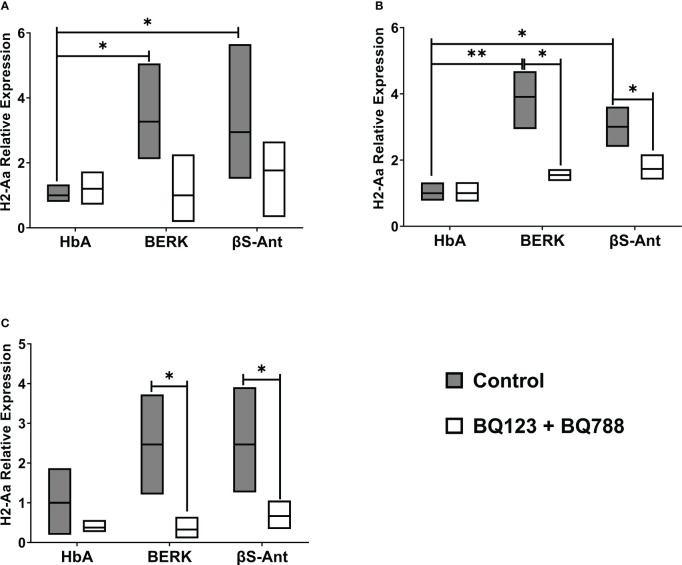
MHC mRNA levels are decreased by *in vivo* treatment with ET-1 receptor antagonists in Sickle Transgenic Mice. MHC class II gene, H2-Aa, was measured in the spleen **(A)**, lung **(B)**, and kidneys **(C)** from BERK, βS-Antilles (βS-Ant) mice and mice expressing human HbA following treatment with or without Endothelin-1 receptor antagonist as described in Methods. Nox plot shows means, SE performed in duplicate in 9 HbA, 7 Berk, and 9 βS-Antilles mice; *p < 0.05, **p < 0.01..

The MHC class II, H2-Aa, gene expression was studied in the HbA mouse model and two SCD mouse models, BERK and βS-Antilles. **Figure 1** shows that the gene expression of H2-Aa is increased in the spleen, lungs, and kidneys of SCD mice compared to the HbA mouse model. In the SCD mouse spleen, we observed increased H2-Aa gene expression of 3.3 ± 1.6 folds in BERK mice and 2.9 ± 1.5 folds in βS-Antilles mice compared to HbA mice (**Figure 1A**). H2-Aa gene expression was likewise significantly increased in the lungs of BERK mice (3.9 ± 0.8 folds) and βS-Antilles mice (3.0 ± 0.6 folds) compared with HbA mice (**Figure 1B**). In addition, we observed an increase of H2-Aa gene expression in the kidneys of BERK mice (2.5 ± 1.3 folds) and βS-Antilles mice (2.5 ± 1.3 folds) compared with HbA mice (**Figure 1C**). H2-Aa gene overexpression was reduced in SCD mouse models treated with ETRA.

### 3.2 MHC expression is stimulated by ET-1

**Figure 2:**
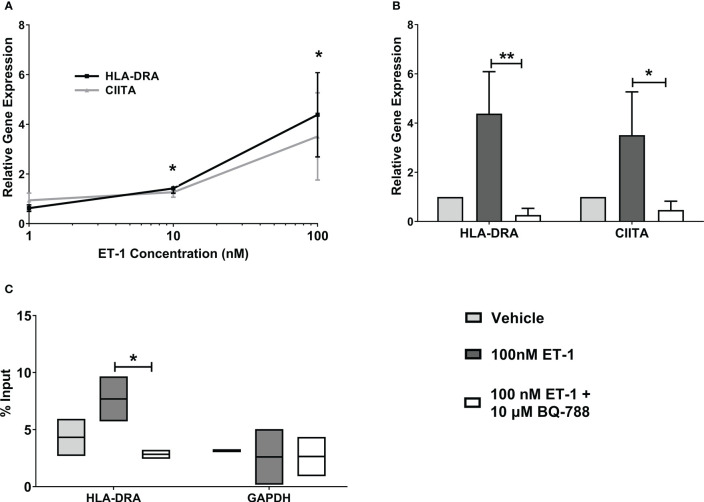
Increased MHC class II expression by ET-1 incubation *in vitro*. **(A)** EA.hy926 endothelial cells were incubated with 1, 10, and 100 nM ET-1. **(B)** EA.hy926 cells were incubated with 100nM ET-1 (dark grey bar) or preincubated with 10µM BQ788, a selective blocker of ET-1 type B receptors (white bar) for 24 h at 37°C and compared to vehicle treatment (light grey bar). Gene expression was quantified by qRT-PCR using TaqMan probes. **(C)** CIITA Chromatin Immunoprecipitation. CIITA is associated with MHC class II promoter in EA.hy926 cells treated with 100 nM ET-1 for 24 hours at 37°C. Lysates were immunoprecipitated with antibodies against CIITA, and DNA was isolated and analyzed by real-time PCR. Values were normalized to the total amount of promoter DNA added to the reaction (input). Input = 5% of total cell lysate. Data represent mean ± SD (n = 3). *p < 0.05, **p < 0.01, 100nM ET-1 versus 100nM ET-1 with 10µM BQ-788..

MHC class II, HLA-DRA, gene expression was significantly increased in human endothelial cells treated with 10nM ET-1 (1.4 ± 0.2 folds) and 100nM ET-1 (4.4 ± 1.7 folds) for 24 hours compared to vehicle-treated cells (**Figure 2A**). Also, we observed that ET-1 stimulated increases in the class II transactivator (CIITA) gene expression, which is a specific transcription factor for MHC class II genes. In addition, pre-treatment of endothelial cells with the endothelin receptor B antagonist, BQ-788, led to reduced ET-1 – stimulated HLA-DRA and CIITA gene expression by about 80-90% (**Figure 2B**). The endothelin receptor A antagonist, BQ123, did not reduce ET-1 – stimulated HLA-DRA and CIITA gene expression significantly (data not shown). To demonstrate HLA-DRA transcription activation, we studied CIITA association with HLA-DRA promoter using Chromatin Immunoprecipitation (ChIP) analyses. We observed increased CIITA association with HLA-DRA promoter following ET-1 treatment, demonstrating HLA-DRA transcription activation (**Figure 2C**).

### 3.3 ET-1 promotes MHC promoter acetylation

**Figure 3:**
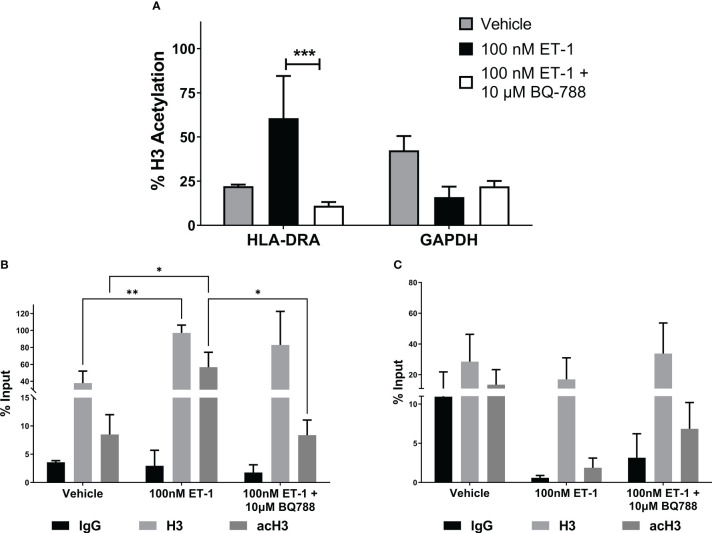
ET-1 induces histone H3 acetylation in MHC class II promoter. EA.hy926 cells were treated with 100 nM ET-1 and 100 nM ET-1 + 10 µM BQ-788 for 24 hours at 37°C. **(A)** Lysates were immunoprecipitated with antibodies against histone H3 and acetyl-K9 histone H3. DNA was isolated and analyzed *via* real-time PCR. Values were normalized to the total amount of promoter DNA added to the reaction (input). Input = 5% of total cell lysate. ***p < 0.001 versus preincubation with BQ-788. **(B)** H3 and acetylated H3 (acH3) chromatin immunoprecipitation for HLA-DRA promoter. **(C)** H3 and acetylated H3 (acH3) chromatin immunoprecipitation for GAPDH promoter. Data represent mean ± SD (n = 3). *p < 0.05, **p < 0.01..

To further demonstrate the genetic activation of HLA-DRA by ET-1, we studied the histone 3 (H3) acetylation in its promoter by ChIP assay in EA.hy926 human endothelial cells. **Figures 3A, B** show that ET-1 treatment in endothelial cells increased acetyl-K9 histone H3 in the HLA-DRA promoter by 38.5% compared to cells without treatment. This effect was abolished in ET-1-stimulated endothelial cells treated with antagonist BQ788, suggesting that ET-1 increases the activation of HLA-DRA. No statistically significant change was observed in the GAPDH promoter (**Figure 3C**).

### 3.4 ET-1 promotes CREB activation and recruitment to MHC promoter

**Figure 4:**
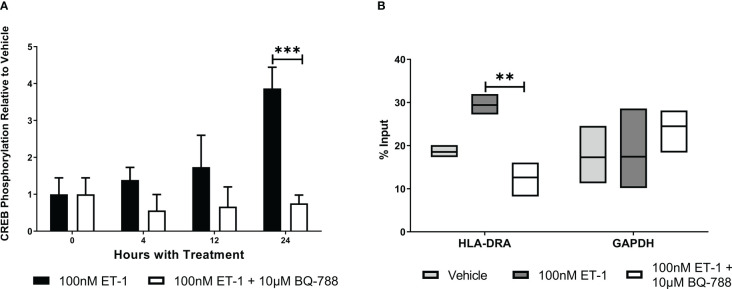
ET-1 stimulates MHC expression by CREB activation. **(A)** The ratio of phospho-CREB (p-CREB) to total CREB in EA.hy926 cells treated with 100 nM ET-1 and 100 nM ET-1 + 10 µM BQ-788 for 24 hours at 37°C. **(B)** CREB chromatin immunoprecipitation. CREB is associated with MHC class II promoter in EA.hy926 cells treated with 100 nM ET-1 and 100 nM ET-1 + 10 µM BQ-788 for 24 hours at 37°C. Lysates were immunoprecipitated with antibodies against CREB, and DNA was isolated and analyzed *via* real-time PCR. Values were normalized to the total amount of promoter DNA added to the reaction (input). Input = 5% of total cell lysate. Data represent mean ± SD (n = 3). **p < 0.01, ***p < 0.001, 100nM ET-1 versus 100nM ET-1 with 10µM BQ-788..

To characterize how ET-1 activates MHC class II transcription, we studied CREB activation. We observed that ET-1 significantly increased CREB phosphorylation (3.9 ± 0.6 folds, **Figure 4**). In addition, CREB activation is associated with HLA-DRA promoter following ET-1 treatment (**Figure 4B**). These results show that CREB activation is one of the mechanisms by which ET-1 stimulates MHC class II gene expression in endothelial cells.

## 4 Discussion

Our results provide evidence that ET-1 is a novel regulator of MHC promoter activity and suggest that ET-1 receptor blockade represents an important therapeutic approach to improve the immune and inflammatory responses and vascular complications in SCD. We show the novel effects of ET-1 on MHC class II regulation in transgenic mouse models of SCD and human endothelial cells. We provide evidence that MHC class II molecules are increased in animal models of SCD but not in mice expressing exclusively human HbA. Our results show that ET-1–stimulated expression of MHC class II molecules is sensitive to blockade of ET-1 receptor B by BQ788 *in vitro* and that *in vivo* MHC class II levels in SCD mice can be reduced by treatment with ET-1 receptor A and B antagonists (ETRAs). Finally, we provide evidence that an important mechanism for ET-1–stimulated MHC expression *via* CREB activation and recruitment to MHC class II promoter sites leads to their acetylation and transcriptional activation.

Increased expression of MHC class II molecules has been proposed as a risk factor for developing inflammatory diseases such as rheumatoid arthritis, multiple sclerosis, and myocardial infarction (41). The novel effects of ET-1 on MHC class II molecules and their increased levels in SCD mouse models suggest that MHC class II molecules contribute to the chronic proinflammatory status in SCD. Thus, MHC class II molecules in SCD may contribute to a vicious cycle of increased inflammatory responses, a chronic proinflammatory state, and increased vaso-occlusive episodes. Consistent with this hypothesis, there is evidence that CD1, an MHC class I and class II related molecule of the β2-microglobulin-associated transmembrane protein family, was shown to be associated with vaso-occlusive episodes and stroke in SCD (42).

MHC class II molecules present antigens to CD4+ T cells. Some studies have reported a decrease in CD4+ T cell numbers in patients with SCD compared to healthy patients (43). However, little is known about T lymphocytes in SCD pathogenesis. It is important to note that continuous antigen presentation may cause T-cell exhaustion (44). Consequently, MHC class II overexpression in SCD may lead to CD4+ T cell exhaustion, making these patients more susceptible to infections.

CREB is a multifunctional transcription factor that plays an essential role in regulating immune responses by activating innate and adaptive immune cells as well as endothelial cells. Growth factors and inflammatory molecules activate CREB leading to the regulation of transcription of genes encoding cytokines containing a cAMP-responsive element such as IL-6 and TNF-α (45). In addition, CREB regulates fetal hemoglobin production in erythroid cells (46). In our study, ET-1 regulated MHC molecules *via* direct effects on CREB activation and recruitment to MHC class II promoter sites. These results confirm and extend a previous report showing that ET-1 activates CREB in neonatal rat cardiac myocytes (47). In addition, we report that ET-1 treatment increases MHC class II promoter activation and the association of CREB and CIITA (MHC class II transcription factors) with the MHC class II promoter DNA. Furthermore, we show that ET-1 treatment increases histone acetylation of the MHC class II promoter, indicating transcriptional activation. Consequently, increased levels of ET-1 in SCD may lead to increased CREB expression in endothelial cells leading to activation of MHC class II transcription factors that contribute to enhanced immune cell activation and further pro-inflammatory responses in SCD.

Multiple etiologies, including bacterial and viral infections, contribute to the pathophysiology of ACS and vaso-occlusion in patients with SCD. There is growing concern that SCD patients are at increased risk for developing ACS and respiratory complications following viral infections, including SARS-CoV-2, through unclear mechanisms (7). Of interest, in patients with SCD, a genetic variant in the MHC class II molecule, MHC HLA-G +3142, showed increased susceptibility to hepatitis C virus infection (48). These results suggest a role for MHC class II in immune responses to viral infectivity in SCD. This is of clinical importance as in adults and children with SCD, influenza, and other viruses, such as H1N1, can lead to ACS and require hospitalization (6). ACS is associated with an inflammatory response that contributes to lung dysfunction and debilitating pain, leading to increased morbidity and mortality in these patients (49, 50). Indeed, there is evidence that markedly elevated circulating pro-inflammatory cytokines such as ET-1, IL-6, vascular cell adhesion molecule-1, and lower levels of cytoprotective factors promote chronic inflammatory responses in the lung and contribute to ACS (51, 52).

There are, however, some limitations to our study. We focused our analyses on MHC class II molecules in human cells, two transgenic SCD mouse models, and a mouse that expresses human HbA. We used the whole organ/tissue RNA and couldn’t determine the effect of ET-1 and its receptor antagonist on specific cell types. Future studies must include both MHC class I and II molecules. In addition, our *in vitro* studies used a well-established human endothelial cell line with caveolin-1 and caveolae –specialized lipid rafts critical for proper signaling compartmentalization in numerous cell types, including endothelial cells (37). This is important as many cell lines lose caveolae in culture (53). However, future studies must include mice and human cells that mediate innate and adaptive immune responses. Also, we focused our studies on the effects of ET-1 as this multifunctional vasoactive pro-inflammatory cytokine is reported to impact the pathophysiology of SCD significantly (20). However, studies on viral infectivity and the impact of additional cytokines on MHC class I and II molecules in SCD models are necessary. Our findings provide a rationale for the development of such studies.

In conclusion, our studies provide *in vitro* and *in vivo* evidence using mouse models of SCD that ET-1 is a novel regulator of MHC class II promoter activity and suggest that ET-1 receptor blockade represents a novel therapeutic approach to improve immune function and inflammatory responses in SCD.

## Data availability statement

The datasets presented in this study can be found in online repositories. The names of the repository/repositories and accession number(s) can be found below: http://dx.doi.org/10.17632/cmghb83rmm.1, 10.17632/cmghb83rmm.1.

## Ethics statement

The animal study was reviewed and approved by Boston Children’s Hospital Animal Care and Use Committees.

## Author contributions

Conceptualization, YI-N, AR, and JRR; methodology, YI-N, AR, and JRR; formal analysis, YI-N, AR, and JRR; investigation, YI-N and AR; writing—original draft preparation, YI-N; writing—review and editing, YI-N, AR, and JRR; supervision, JRR; funding acquisition, AR and YI-N. All authors contributed to the article and approved the submitted version.
